# Falls and patterns of physical activity participation over 18 years in the Australian Longitudinal Study on Women’s Health

**DOI:** 10.1136/bjsports-2024-108262

**Published:** 2024-07-24

**Authors:** Wing S Kwok, Saman Khalatbari-Soltani, Xenia Dolja-Gore, Julie Byles, Juliana S Oliveira, Marina B Pinheiro, Anne Tiedemann, Catherine Sherrington

**Affiliations:** 1 The University of Sydney Institute for Musculoskeletal Health, Sydney, New South Wales, Australia; 2 School of Public Health, The University of Sydney, Sydney, New South Wales, Australia; 3 ARC Centre of Excellence in Population Aging Research (CEPAR), University of Sydney, Sydney, New South Wales, Australia; 4 The University of Newcastle, Callaghan, New South Wales, Australia

**Keywords:** Accidental Falls, Physical activity, Women, Longitudinal Studies, Prospective Studies

## Abstract

**Objective:**

To explore the relationship between long-term physical activity (PA) participation and falls.

**Methods:**

Participants in the Australian Longitudinal Study of Women’s Health born 1946–1951 self-reported amounts of PA every 3 years since 1998 (mean age: 54 years, n=11 796). Latent class analysis described profiles of self-reported PA participation over 18 years. Associations between patterns of PA participation and self-reported falls measured in 2019 were examined using multinomial logistic regression adjusted for directed-acyclic graph-informed potential confounders, with the highly active group as the reference category.

**Results:**

Women were grouped into five PA participation profiles. Compared with consistently highly active patterns (maintaining ≥300 min/week, 22%), consistently lower levels of PA<100 min/week (18%), consistently some PA<150 min/week (18%) and decreasing PA but maintaining≥150 min/week (n=3540, 30%) had higher odds of non-injurious falls (odds Ratio_lower level_ (OR): 1.59, 95% CI 1.29 to 1.97; OR_some PA_: 1.27, 95% CI 1.04 to 1.55; OR_decreasing activity_:1.29, 95% CI 1.02 to 1.63) and injurious falls (OR_low level_: 1.32, 95% CI 1.06 to 1.64; OR_some PA_: 1.27, 95% CI 1.04 to 1.54; OR_decreasing activity_: 1.47, 95% CI 1.18 to 1.83). No association was found between increasing PA (≥150 min/week, 11%) for non-injurious (OR 1.07, 95% CI 0.89 to 1.29) and injurious falls (OR 1.07, 95% CI 0.90 to 1.29). After adjusting for potential confounders, consistently lower levels of PA remained associated with increased non-injurious falls odds (OR_1998 survey_: 1.40, 95% CI 1.11 to 1.77; OR_2016 survey_: 1.35, 95% CI 1.07 to 1.71).

**Conclusion:**

The increased odds of falls among women with consistently lower levels of PA over 18 years supports ongoing participation of 150+ min/week of PA.

**WHAT IS ALREADY KNOWN ON THIS TOPIC**

- Structured exercise has been found to reduce falls in trials. However, the follow-up periods in trials are usually short.
- One previous study looked at the associations between long-term physical activity participation from mid-life to older age and falls regardless of injuries. No previous study has examined the associations between long-term physical activity participation from mid-life to older age and injurious falls in older people.

**WHAT THIS STUDY ADDS**

- Five distinct patterns of physical activity participation were found.
- Women reporting consistently low levels of physical activity over 18 years experienced an increased risk of subsequent falls.

**HOW THIS STUDY MIGHT AFFECT RESEARCH, PRACTICE OR POLICY**

- The finding supports promotion of ongoing participation in more than 150 min/week of physical activity for falls prevention.

## Introduction

Falls are a recurring problem for older people as more than one in three older adults experiences at least one fall each year.^1^ The incidence of falls increases with age^1^ and is higher in women than men.^2^ Falls and fall-related injuries can result in detrimental outcomes including hospitalisation, activity avoidance, loss of independence^3^ and are one of the leading risk factors for deaths and disability in Australia.^4^

The WHO 2020 Guidelines on Physical Activity and Sedentary Behaviour recommends older adults undertake 150–300 min of moderate-intensity physical activity (PA) per week.^5 6^ The guidelines also recommend that older adults should engage in structured exercise (ie, a type of PA) that targets balance and function to prevent falls.^5 7^ However, as follow-up periods in trials are usually short,^7^ cohort studies are better placed to explore longer-term relationships between PA and falls. A cohort study of women (n=7139) indeed found that participation in PA at the recommended level by the WHO or above was associated with reduced falls 3 years later.^8^ However, the association between longer-term PA participation and falls is yet to be examined.

PA participation often changes during mid-life because of life-transition events, such as diagnosis of serious diseases, retirement or menopausal among women.^9 10^ Some studies estimated patterns of PA participation across life stages to understand differences in behaviour and the impact of different PA participation patterns on health in different age groups, including people in mid-life and older age.^11^ Of the eight studies included in a systematic review that described different PA participation patterns in late middle and older age,^11^ three investigated the relationship between PA participation patterns and subsequent outcomes.^12–14^ These studies found that (1) among people in the UK (aged 35–55 years at baseline, n=6825), physically inactive patterns over 20 years were associated with increased disability at later life^12^; (2) women in the USA (aged 42–52 years at baseline, n=3302) with the highest PA participation pattern had better physical functioning performance in later life^13^ and (3) older women in the USA (aged 70–79 years at baseline, n=433) with patterns of declining PA or who were always inactive had increased risk of death.^14^

As for falls, a cohort study in the USA looked at moderate-vigorous intensity PA participation patterns and subsequent falls risk (mean (range) age: 54 (45–64) years, n=15 792), reported four distinct PA participation patterns and found that decreasing moderate-vigorous PA across mid-life was associated with an increased fall risk (falls regardless of injuries).^15^ However, this study recorded PA participation for 24 years by using three time points (ie, baseline, 6-year and 24-year postbaseline survey). Data collection at just three time points over this long follow-up time may lead to misclassification and also limit the ability to accurately construct patterns of PA participation.^15^ As amount of PA participation or health status (eg, physical function)^16^ changes over time across lifespan, the use of more follow-up surveys and with consistent period of follow-up could lead to refinement in identifying distinct patterns of amounts of PA participation. To our knowledge, no study has investigated the association between long-term PA participation and falls with and without injuries.

Data from the Australian Longitudinal Study on Women’s Health (ALSWH) on PA participation over 18 years with consistent length of follow-up between surveys provide a unique opportunity to understand the longitudinal pattern of amounts of PA participation from mid-life to older age. We aimed to use these data to:

- Identify and describe different patterns of amounts of PA participation in women from mid-life to older age.
- Examine the association between patterns of amounts of PA participation, and falls and injurious falls.

## Method

### Data source

The ALSWH is an ongoing population-based study.^17^ It began in 1996 with samples of Australian women born 1921–26 (aged 70–75 years at baseline), 1946–51 (aged 45–50) and 1973–78 (aged 18–23), randomly selected from the Medicare—national health insurance database, which included all Australian citizens and permanent residents regardless of income.^18^ Rural and remote areas were intentionally oversampled to ensure a good representation of these women.^18^ Data for race and ethnicity were unfortunately not collected in 1996. There were no exclusion criteria when recruiting participants.

The current study used data from the 1946–1951 born cohort. Participants responded to follow-up surveys every 3 years after survey 2 in 1998. The 1946–1951 cohort in the ALSWH was comparable to the corresponding census data, although more women in the ALSWH were employed in 1996.^18^ The majority of the participants were born in Australia (70%), reported either being married or in a de facto relationship (81%) and were employed (73%) in 1996.^18 19^ To identify patterns of amount of PA participation, we used the data from 1998 (survey 2, aged 47–52) to 2016 (survey 8, aged 65–70 years) and to examine the relationship between PA participation patterns and falls, we used the outcome of falls in 2019 (survey 9, aged 68–73).

### Exposure: PA

Self-reported PA of seven surveys from survey 2 (1998) to survey 8 (2016) was measured using the validated self-reported version of Active Australia National Physical Activity survey.^20^ In this study, we used the amount of weekly PA participation, which was based on the total duration of the three types of PA including walking briskly, moderate and vigorous leisure activity. Weekly PA participation was categorised into 0, 1 to <150, 150 to <300 and ≥300 min, according to the recommended targeted duration of moderate-intensity activity by WHO.^6^ The categorisation of PA according to the guideline recommended levels sought to enhance interpretability of results. Table 1 shows the PA questions asked in the survey.

**Table 1:** Physical activity and falls collected in the Australian Longitudinal Study on Women’s Health (ALSWH)

Variable	Exposure and outcome collected in the ALSWH and how they were used in this study
Physical activity* (exposure)	Questions asked in the ALSWH (surveys 2–8) How many times did you do each type of activity last week? (only counted the number of times when the activity lasted for 10 min or more). Walking briskly (for recreation or exercise or to get from place to place). Moderate leisure activity (social tennis, moderate exercise classes, recreational swimming, dancing). Vigorous leisure activity (that makes one breath harder or puff and pant like aerobics, vigorous cycling, running, swimming). If you add up all the times you spent in each activity last week, how much time did you spend altogether doing each type of activity? Methods of categorising physical activity participation in this study The total amount of weekly physical activity participation was the sum of the above-mentioned three types of activities and was categorised into: 0, 1 to <150, 150 to <300 and ≥300 min
Falls (outcome)	Questions asked in the ALSWH†: In the last twelve months, have you Had a fall to the ground? Been injured as a result of a fall? Needed to seek medical attention for an injury from a fall? Methods of categorising falls in this study Participants were coded as having non-injurious falls if they reported a fall to the ground without a positive response to the second or third question about injury because of a fall and had injurious falls if they gave a positive response to the first and the second and/or third questions. The outcome was divided into three levels: no falls (reference group), non-injurious falls and injurious falls.

*To ensure the consistency of the questions asked about PA across all surveys, PA collected in survey 1 was not used as participants were asked to report the activity lasted for 20 min or more.

†Falls questions were included from survey 4 (2004).

PA, physical activity.

### Outcomes: non-injurious and injurious falls

Participants responded to questions about falls with and without injuries (table 1) from survey 4 (2004) to survey 9 (2019). Using the approach of temporal procedure of exposure over outcome, we used fall data in survey 9. In this study, the outcome was divided into three levels: no falls (reference group), non-injurious falls and injurious falls.

### Potential demographic and clinical confounders

Using expert knowledge in the field of PA and falls, and available literature, the authors identified potential confounders for the causal association between PA and falls. Potential confounders were grouped together within nodes when they shared similar concepts/themes. The authors constructed a directed acyclic graph (DAG) (online supplemental figure 1) using open-access software DAGitty V.3.0, before any analysis was performed.^21^ The variables considered in the DAG are described in online supplemental table 1. A DAG is a causal path diagram that forms a hypothesised theoretical framework to enable the selection of a minimally sufficient set of variables considered to confound the exposure-and-outcome relationship.^22 23^ The identified minimal sufficient set of covariates minimises the confounding bias while not introducing other type of biases (eg, overadjustment bias and collider bias).^23 24^ The DAG identified two different models for adjustment: (1) area of residence, defined by the Accessibility Remoteness Index of Australia scale (ARIA+), body mass index (BMI), number of health conditions, education level and ability to manage income (ie, based on the question ‘how do you manage on the income you have available’) and (2) ARIA+, BMI, the number of health conditions and perceived stress (scale between 0 (not at all stressed) to 4 (extremely stressed)).

10.1136/bjsports-2024-108262.supp1 Supplementary data

### Statistical analysis

#### Pattern of amounts of PA participation

Repeated-measures latent class analysis was used to classify participants into mutually exclusive categories based on patterns of PA amounts at each survey, and the probability of class membership.^25^ Participants who responded to PA questions in at least one survey between survey 2 (1998) and survey 8 (2016) were included, as latent class analysis takes into account incomplete exposure.^26^ To ensure that the measurement of exposure preceded the measurement of outcome,^27^ we did not include PA of survey 9 (2019) in the repeated-measures latent class analysis as falls in survey nine was used. One-cluster to six-cluster models were tested and the fit for the number of clusters selected was based on: Akaike information criterion (AIC); Bayesian information criterion (BIC) (smaller AIC and BIC indicate better fit to the data); entropy values (larger entropy value indicates better classification accuracy).^26^ A line graph of fit statistics was created to visualise the point at which the decrease in fit statistics became less evident. Item response probabilities of the amounts of PA participation pattern were presented graphically. We also considered the theoretical and clinical meaningfulness of profile memberships.^26^ The Guideline for Reporting on Latent Trajectory Studies^28^ was followed (online supplemental table 2). Descriptive statistics were used to summarise the characteristics of each identified latent class membership.

To ensure stability and robustness of the identified latent classes and the reliability of findings, a separate repeated-measures latent class analysis was run with the sample size after excluding participants with missing outcome (ie, samples included in multinomial logistic regression). Pearson correlation analysis was used to examine the association between the class assigned using the initial sample size and sample after excluding participants with missing outcome.

#### Association between latent class memberships of PA and falls

To avoid multiple testing while examining the association between latent class memberships of PA participation and falls within one analysis, multinomial logistic regression was used. Given the clear benefits of the high level of PA and low PA participation globally, we sought to explore the relationships between different patterns of PA and falls. Therefore, latent class membership with the highly active group from mid-life to older age was chosen as the reference category. Effect estimates were presented as ORs with their 95% CIs and were adjusted for potential confounders. To provide a structured and holistic approach to understanding the associations, potential confounders in survey 2 (1998) and survey 8 (2016) were used separately for both models identified by DAG. Sensitivity analyses were conducted by examining the associations of latent class memberships of PA participation and falls using complete-case analysis when adjusting for potential covariates for both DAG-identified models separately in survey 2 and survey 8 (ie, only included participants with no missing data for falls and all covariates in the models). All statistical analyses were undertaken by using SAS V.9.4 (×64), SAS Institute.

### Patient and public involvement

Patient or members of the public were not involved in the design or conduct of this current study.

### Equity, diversity and inclusion statement

In the ALSWH, women from different geographic and socioeconomic backgrounds were included. Rural and remote areas were intentionally oversampled to ensure a good representation of these women.^18^ Our study addressed for potential geographic and socioeconomic differences and included these factors as potential covariates. Members of our research team included senior, mid and early-career academics and clinician.

## Results

**Figure 1:**
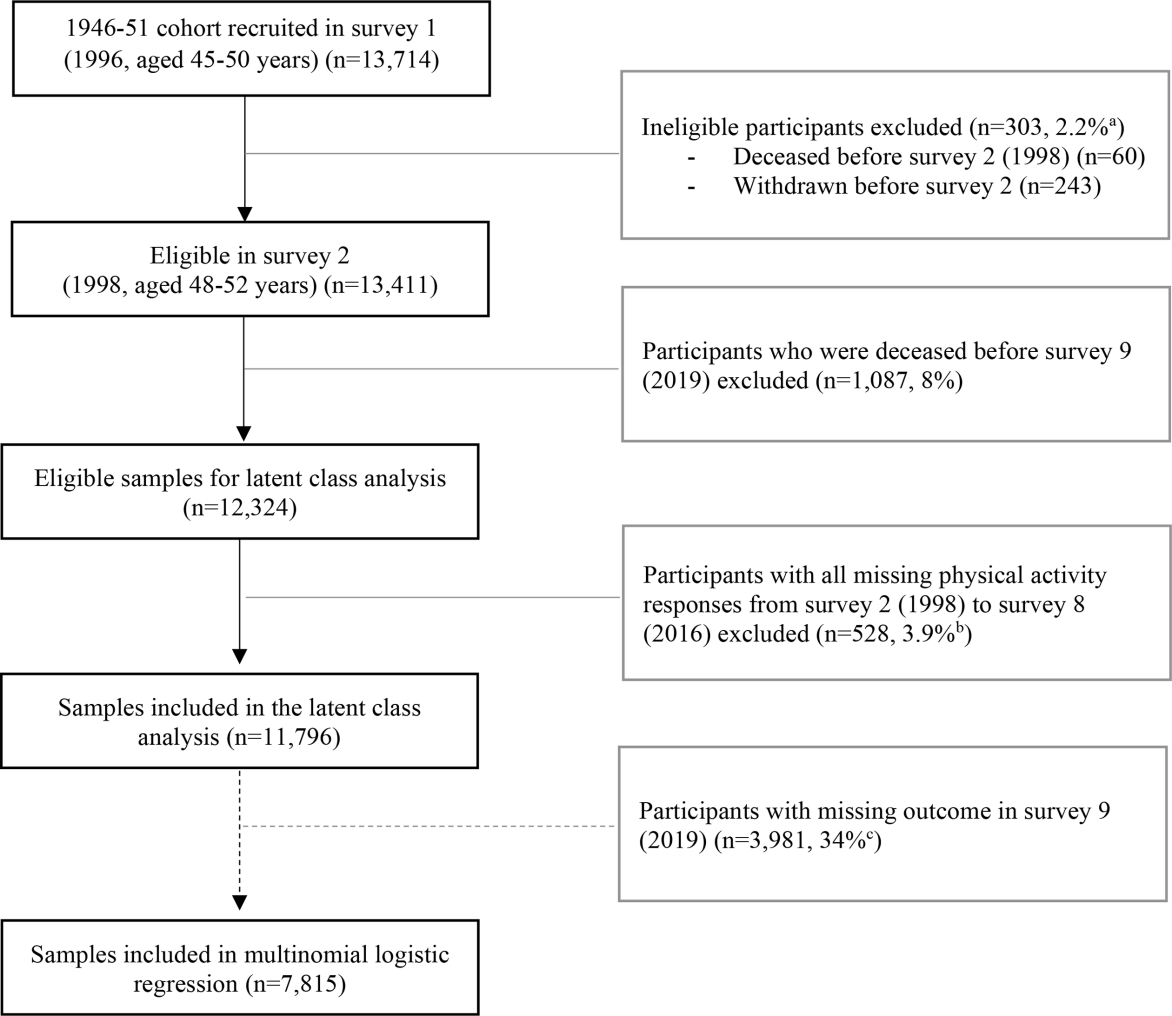
Sample selection flow chart of the 1946–1951 born women in the Australian Longitudinal Study of Women’s Health.^a^The percentage was calculated based on the number of participants who were recruited in survey 1 (n=13,714). ^b^The percentage was calculated based on the number of participants who were recruited in survey 2 (n=13,411). ^c^The percentage was calculated based on the number of participants included in the latent class analysis (n=11,796).

ALSWH participants were considered ineligible for analyses if they were deceased (n=60) or withdrew from the study (n=243) before survey 2, leaving 13 411 eligible participants (figure 1). As this study aimed to explore the associations between PA participation patterns and self-reported falls in survey 9 (2019), participants who were deceased by survey 9 (n=1087) were excluded. A further 528 women were excluded due to missing responses in all PA questions, leaving 11 796 women in the latent class analysis (figure 1). Table 2 shows the characteristics of the included participants. About two-thirds of the participants had school or higher school certificate education (68%) and had ≤1 health condition (63%) at baseline (survey 2). The majority (73%) of participants lived in either major cities or inner regional areas. Less than half of the participants had BMI≥18.5 to <25 kg/m^2^ (44%) and only half of the participants reported being able to manage income (responded as ‘not too bad’ (37%) or ‘easy’ (14%)). The mean reported stress was 0.62 points (SD 0.5) on the 5-point ALSWH perceived stress scale.

**Table 2:** Participant characteristics (at survey 2 and survey 8) overall and by patterns of amounts of physical activity (PA) participation

Characteristics	Total n=11 796		Consistently lower level of PA n=2143		Consistently some PA n=2147		Decreasing PA n=1345		Increasing PA n=3540		Consistently highly active n=2621	
	Survey 2	Survey 8	Survey 2	Survey 8	Survey 2	Survey 8	Survey 2	Survey 8	Survey 2	Survey 8	Survey 2	Survey 8
Age, years, mean (SD)	49.5 (1.5)	67.7 (1.5)	49.5 (1.5)	67.7 (1.4)	49.5 (1.5)	67.7 (1.5)	49.5 (1.5)	67.8 (1.5)	49.5 (1.5)	67.7 (1.5)	49.5 (1.4)	67.8 (1.4)
Smoking status, n (%)												
Never smoker	6106 (52)	5335 (45)	958 (45)	711 (33)	1180 (55)	1041 (48)	637 (47)	623 (46)	1945 (55)	1699 (48)	1386 (53)	1261 (48)
Ex-smoker	2834 (24)	2620 (22)	453 (21)	401 (19)	456 (21)	458 (21)	389 (29)	379 (28)	871 (25)	784 (22)	665 (25)	598 (23)
Smoker	1670 (14)	424 (4)	461 (22)	127 (6)	266 (13)	68 (3)	205 (15)	67 (5)	417 (11)	86 (2)	321 (12)	66 (3)
Missing	1186 (10)	3417 (29)	271 (13)	904 (42)	245 (11)	580 (27)	114 (8)	276 (21)	307 (9)	971 (27)	249 (10)	686 (26)
Menopausal Status, n (%)*												
Premenopausal	2569 (22)	–	386 (18)	–	468 (22)	–	276 (21)	–	808 (23)	–	631 (24)	–
Perimenopausal	2749 (23)	–	434 (20)	–	494 (23)	–	300 (22)	–	883 (25)	–	638 (24)	–
Postmenopausal/surgical menopause	4129 (35)	–	876 (41)	–	754 (35)	–	497 (37)	–	1143 (32)	–	859 (33)	–
HRT use or OCP use	1669 (14)	–	287 (13)	–	300 (14)	–	198 (15)	–	546 (15)	–	338 (13)	–
Missing	680 (6)	–	160 (7)	–	131 (6)	–	74 (6)	–	160 (5)	–	155 (6)	–
Education, n (%)† ‡												
No formal education	1984 (17)	1868 (16)	620 (29)	608 (28)	370 (17)	330 (15)	245 (18)	229 (17)	446 (13)	412 (12)	303 (12)	289 (11)
School/intermediate certificate/higher school/ leaving certificate	7987 (68)	7771 (66)	1360 (63)	1354 (63)	1434 (67)	1409 (66)	933 (69)	911 (68)	2417 (68)	2338 (66)	1843 (70)	1759 (67)
University degrees or above	1725 (15)	2123 (18)	142 (7)	170 (8)	327 (15)	405 (19)	155 (12)	203 (15)	649 (18)	779 (22)	452 (17)	566 (22)
Missing	100 (1)	34 (0.3)	21 (1)	11 (0.5)	16 (1)	*	12 (1)	*	28 (1)	11 (0.3)	23 (1)	*
Ability to manage income, n (%)‡												
Impossible	206 (2)	124 (1)	73 (3)	42 (2)	39 (2)	28 (1)	25 (2)	11 (1)	48 (1)	23 (1)	21 (1)	20 (1)
Always difficult	1323 (11)	639 (5)	350 (16)	192 (9)	222 (10)	118 (6)	168 (12)	113 (8)	369 (10)	125 (4)	214 (8)	91 (3)
Sometimes difficult	3067 (26)	1690 (14)	579 (27)	313 (15)	617 (29)	350 (16)	375 (28)	226 (17)	883 (25)	479 (14)	613 (23)	322 (12)
Not too bad	4340 (37)	4064 (34)	664 (31)	525 (25)	778 (36)	760 (35)	481 (36)	522 (39)	1392 (39)	1299 (37)	1025 (39)	958 (37)
Easy	1632 (14)	1842 (16)	194 (9)	162 (8)	243 (11)	305 (14)	174 (13)	192 (14)	532 (15)	640 (18)	489 (19)	543 (21)
Missing	1228 (10)	3437(29)	283 (13)	909 (42)	248 (12)	586 (27)	122 (9)	281 (21)	316 (9)	974 (28)	259 (10)	687 (26)
Location (ARIA+), n (%)† ‡												
Major cities	3938 (33)	3450 (29)	652 (30)	444 (21)	712 (33)	661 (31)	415 (31)	398 (30)	1285 (36)	1135 (32)	874 (33)	812 (31)
Inner regional	4770 (40)	3570 (30)	852 (40)	555 (26)	872 (41)	659 (31)	541 (40)	449 (33)	1371 (39)	1063 (30)	1134 (43)	844 (32)
Outer regional, remote and very remote	3087 (26)	1813 (15)	639 (30)	333 (16)	563 (26)	349 (16	389 (29)	272 (20)	884 (25)	505 (14)	612 (23)	354 (14)
Missing	*	2963 (25)	*	811 (38)	*	478 (22)	*	226 (17)	*	837 (24)	*	611 (23)
Body mass index, n (%)‡												
<18.5 kg/m^2^	146 (1)	116 (1)	21 (1)	12 (1)	22 (1)	21 (1)	13 (1)	12 (1)	43 (1)	37 (1)	47 (2)	34 (1)
≥18.5 to <25 kg/m^2^	5247 (44)	2934 (25)	648 (30)	251 (12)	838 (39)	444 (21)	575 (43)	304 (23)	1750 (49)	1015 (29)	1436 (55)	920 (35)
≥25 to <25 kg/m^2^	3430 (29)	2811 (24)	620 (29)	354 (17)	640 (30)	533 (25)	420 (31)	353 (26)	1058 (30)	947 (27)	692 (26)	624 (24)
≥30 kg/m^2^	2211 (19)	2581 (22)	645 (30)	607 (28)	494 (23)	601 (28)	260 (19)	397 (30)	504 (14)	607 (17)	308 (12)	369 (14)
Missing	762 (6)	3354 (28)	209 (10)	919 (43)	153 (7)	548 (26)	77 (6)	279 (21)	185 (5)	934 (26)	138 (5)	674 (26)
Number of health conditions, n (%)‡												
Nil	3934 (33)	2541 (22)	634 (30)	284 (13)	650 (30)	426 (20)	447 (33)	282 (21)	1241 (35)	844 (24)	962 (37)	705 (27)
1	3501 (30)	2758 (23)	579 (27)	367 (17)	629 (29)	536 (25)	383 (28)	330 (25)	1113 (31)	865 (24)	797 (30)	660 (25)
2	2079 (18)	1737 (15)	410 (19)	267 (12)	413 (19)	344 (16)	250 (19)	240 (18)	576 (16)	512 (14)	430 (16)	374 (14)
≥3	1651 (14)	1421 (12)	373 (17)	341 (17)	333 (16)	278 (13)	198 (15)	231 (17)	457 (14)	365 (11)	290 (12)	206 (8)
Missing	631 (5)	3339 (28)	147 (7)	884 (41)	122 (6)	563 (26)	67 (5)	262 (19)	153 (4)	954 (27)	142 (5)	676 (26)
Report of poor memory, n (%)§												
Never/rarely	–	4948 (42)	–	656 (31)	–	870 (41)	–	602 (45)	–	1599 (45)	–	1221 (47)
Sometimes/often	–	3458 (29)	–	591 (28)	–	704 (33)	–	471 (35)	–	972 (27)	–	720 (27)
Missing	–	3390 (29)	–	896 (42)	–	573 (27)	–	272 (20)	–	969 (27)	–	680 (26)
Perceived stress score, mean (SD)‡ ¶	0.62 (0.50)	0.48 (0.41)	0.69 (0.55)	0.58 (0.49)	0.64 (0.50)	0.51 (0.41)	0.63 (0.50)	0.52 (0.43)	0.59 (0.48)	0.45 (0.37)	0.56 (0.47)	0.43 (0.39)
SF-36 Physical function, mean (SD)**	85 (18)	77 (22)	76 (24)	59 (27)	83 (18)	73 (21)	85 (17)	71 (23)	87 (15)	82 (16)	90 (14)	85 (16)
SF-36 Mental Health, mean (SD)††	74 (18)	78 (17)	69 (20)	71 (20)	72 (18)	76 (16)	74 (18)	75 (18)	75 (17)	80 (15)	78 (16)	81 (14)

Participant characteristics of each cluster in the five-cluster model were described using survey 2 (1998) and survey 8 (2016) in the Australian Longitudinal Study of Women’s Health (ALSWH).

Mean (SD) was used for continuous variables and n (%) for categorical variables.

*Menopausal status data not available in survey 8.

†For participants confidentiality, missing data with a small sample size (n<10) are presented with *.

‡Variables identified from the directed acyclic graph as potential confounders.

§Participants described whether they had reported poor memory in the past 12 months.

¶Stress score measured with the ALSWH perceived stress scale was presented according to the data available (sample size included from left to right column: n=10 622; n=8410; n=1875; n=1245; n=1908; n=1572; n=1229; n=1077; n=3,241; n=2578; n=2369; n=1938).

**SF-36 Physical function was presented according to the data available (sample size included from left to right column SF-36 Physical function: n=11 096; n=8421; n=1982; n=1248; n=2016; n=1580; n=1270; n=1076; n=3367; n=2581; n=2461; n=1938).

††SF-36 Mental Health measured was presented according to the data available (sample size included from left to right column SF-36 Mental Health: n=11 131; n=8421; n=1992; n=1244; n=2020; n=1579; n=1276; n=1079; n=3377; n=2581; n=2466; n=1938).

ARIA, Accessibility Remoteness Index of Australia;HRT, hormone replacement therapy; OCP, oral contraceptive; PA, physical activity; SF-36, Short Form-36.

For the multinomial logistic regression, a further 3981 women were excluded due to missing responses about falls in survey 9 (2019), leaving 7815 women for the analyses (figure 1). Characteristics of participants included in the multinomial logistic regression and those excluded from these analyses due to missing falls are shown in online supplemental table 3. Those excluded from the multinomial logistic regression because of missing response about falls in survey 9 had a greater proportion of missing responses in BMI (11%), no formal education (24%), and a lower proportion reported ‘not too bad’ (32%) or ‘easy’ (10%) in the ability of managing on income (online supplemental table 3).

### Patterns of amounts of PA participation

The distribution of PA participation from survey 2 to survey 9 is shown in online supplemental table 4. The model fit statistics (online supplemental table 5) and line graphs (online supplemental figure 2) were compared for fit. The line graphs showed the decrease in fit indices became flat after five clusters and hence only 1–6 latent clusters models were fit. The rate of decrease in fit statistics (online supplemental figure 2) became less pronounced beyond the five-cluster model, indicating the additional cluster would not sufficiently improve model fit to justify the additional complexity. Theoretical and clinical meaningfulness and description of potentially distinct patterns were also considered. The classification with five clusters was determined to be the best model. The assignment of classes from repeated-measure latent class analysis using sample size of n=11796 and that of n=7815 were similar (online supplemental table 6), with Pearson’s correlation of 0.94.

**Figure 2:**
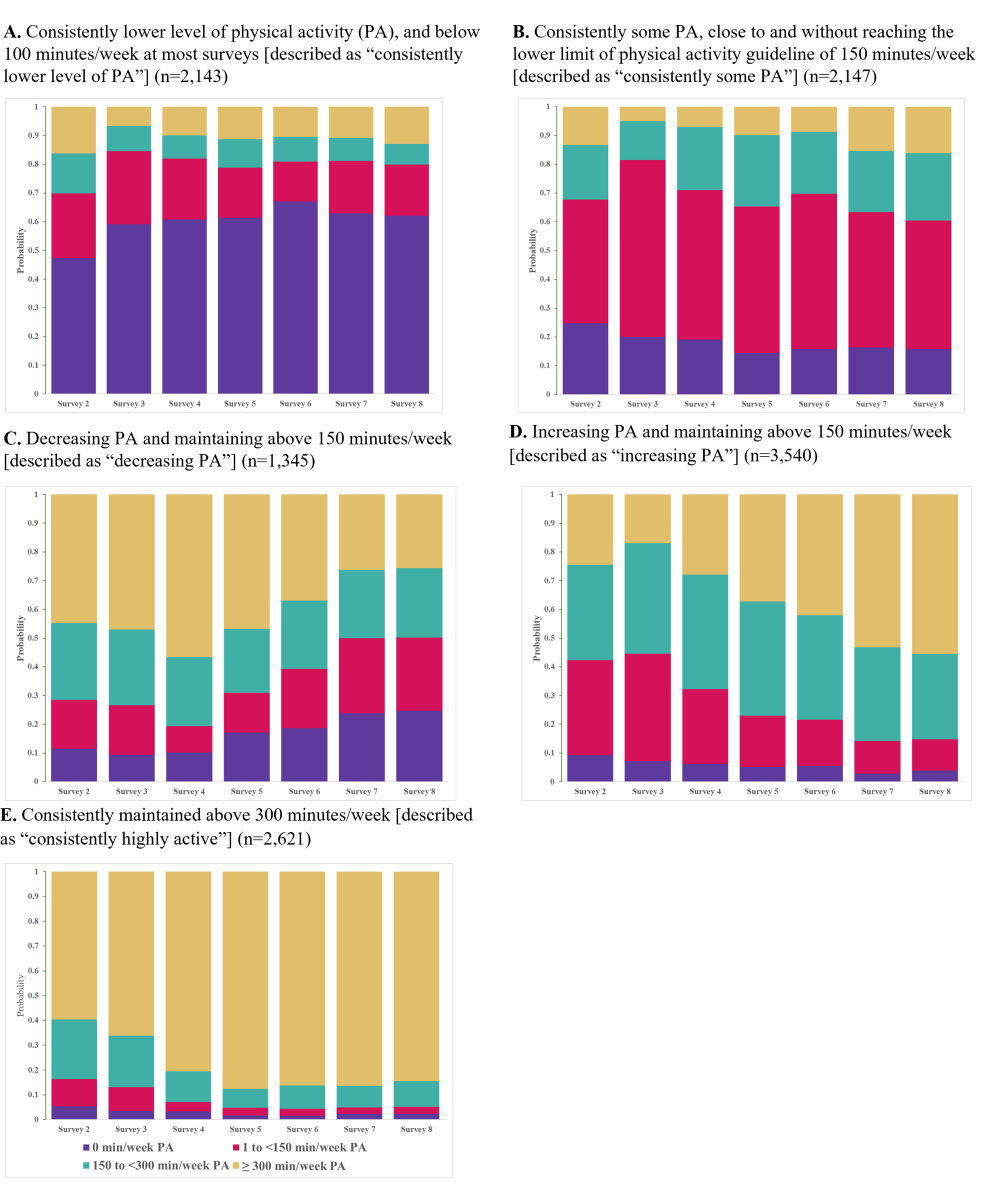
Five different patterns of amount of physical activity participation from middle age to older age in the Australian Longitudinal Study on Women’s Health. PA, physical activity.

**Figure 3:**
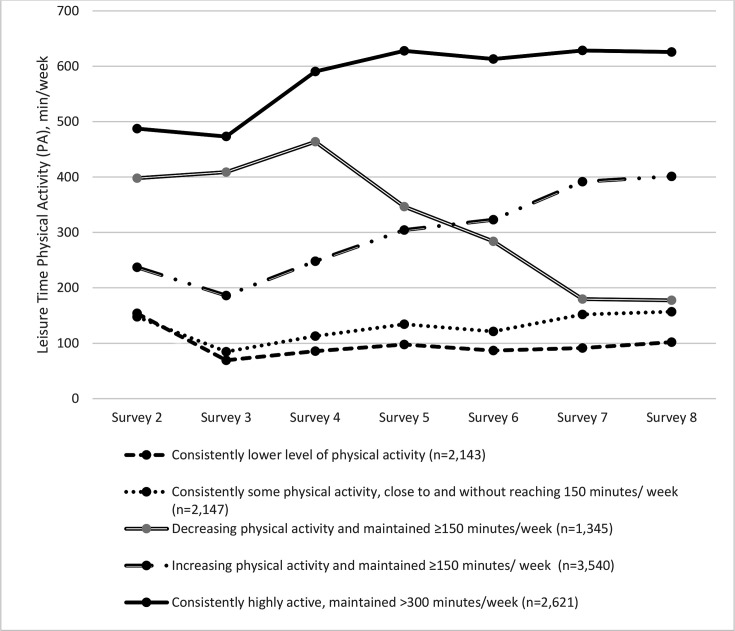
Average amount of physical activity per week performed by the five patterns of amounts of physical activity participation (n=11 796).

Figure 2 shows the probability of participants’ distribution of PA participation for each survey in the five-cluster model. The five mutually exclusive latent class memberships of PA participation over 18-year span reflect consistently lower level of PA (identified as below 100 min/week PA at most surveys) (n=2143, 18%), consistently some PA (identified as close to and without reaching the lower limit of PA guideline of 150 min/week) (n=2147, 18%), decreasing PA (identified as decrease in PA and maintaining above 150 min/week) (n=1345, 11%), increasing PA (identified as increasing PA and maintaining above 150 min/week) (n=3540, 30%) and consistently highly active (identified as consistently maintained≥300 min/week of PA) (n=2621, 22%) (figure 3). Table 1 shows the characteristics of included participants by the five latent class memberships of amounts of PA participation.

Overall, the area of residence was similar among participants with different latent class memberships of PA participation. Greater proportions of participants who consistently had lower level of PA had no formal education (29% at survey 2), were obese (30%) and had three or more health conditions (17%), compared with participants with other four latent class memberships of PA participation. Similar proportions of the participants who had some PA or decreasing PA, increasing PA and were consistently highly active had a university degree or above (range from 12% of those with decreasing PA to 18% among those with increasing PA). Nearly, half of the participants reported ‘not too bad’ or ‘easy’ to manage income (49% for both some PA and decreasing PA). About half of the participants who had some PA or decreasing PA were overweight or obese. Compared with participants who consistently had lower level of PA, some PA or decreasing PA, greater proportions of participants with increasing PA and consistently highly active profiles had normal weight (increasing PA: 49%, consistently highly active: 55%) and reported nil health condition (increasing PA: 35%, consistently highly active: 37%). The perceived stress score on the 5-point ALSWH perceived stress scale is similar across the participants with different latent class memberships of PA participation (table 1). After excluding participants with missing response in falls (in survey 9) (n=3981) for the regression analysis, the characteristics of each PA participation profile remained similar (online supplemental table 3).

### Association between latent class memberships of PA participation and falls

Overall, 1101 (14%) women reported non-injurious and 1150 (15%) reported injurious falls in 2019 (online supplemental figure 3 shows the proportion of fallers from surveys 4–9). Compared with the consistently highly active profile, consistently lower level of PA, consistently some PA and decreasing PA profiles were all associated with higher odds of non-injurious and injurious falls. No differences were found between the increasing PA profile and the consistently highly active profile for non-injurious or injurious falls (online supplemental figure 4).

**Figure 4:**
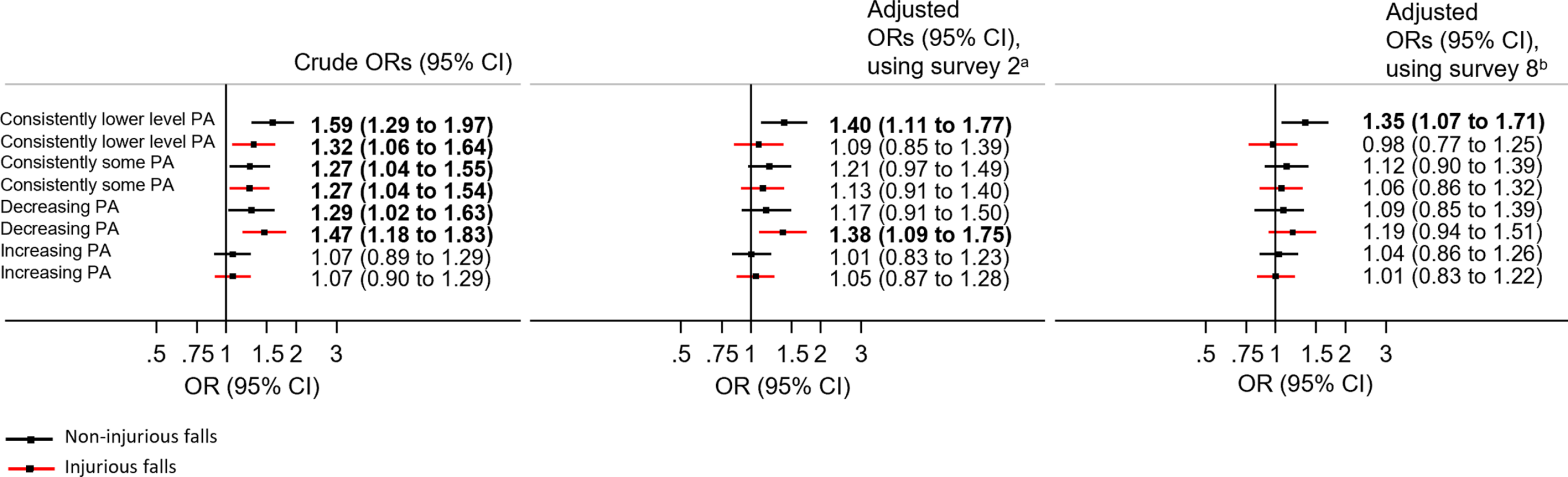
Associations between patterns of amounts of physical activity participation and subsequent falls and injurious falls. Association between different subgroups of physical activity participation pattern from middle age (survey 2, 1998, aged 47–52 years) to older age (survey 8, 2016, aged 65–70 years) and subsequent falls and injurious falls (aged 68–73 years) using multinomial logistic regression and presented in ORs and 95% CIs. Consistently highly active was used as the reference group. Crude model (n=7815). ^a^Adjusted for Accessibility Remoteness Index of Australia scale (ARIA+), body mass index, the number of health conditions, education level and ability to manage income collected in survey 2 (n=7062). ^b^Adjusted for Accessibility Remoteness Index of Australia scale (ARIA+), body mass index, number of health conditions, education level and ability to manage income collected in survey 8 (n=7140). Confounders in survey 2 and survey 8 were adjusted in separate models.

After adjusting for ARIA+, BMI, the number of health conditions, education level and ability to manage income collected at survey 2 or survey 8, only consistently lower level of PA was associated with higher non-injurious falls (figure 4). Decreasing PA was associated with a higher odd of injurious falls after adjusting for covariates at survey 2 (1.38, 95% CI 1.09 to 1.75), but the association was no longer significant after adjusting for the same set of covariates collected at survey 8 (1.19, 95% CI 0.94 to 1.51) (figure 4).

Association between the different latent class memberships of PA participation patterns and subsequent odds of non-injurious and injurious falls were similar after adjusting for the second minimally sufficient set of covariates suggested by the DAG (ARIA+, BMI, the number of health conditions and mean perceived stress) at both survey 2 and survey 8 (online supplemental figure 5). The odds of non-injurious falls for those with low level of PA were 1.41 (95% CI 1.12 to 1.77), for some PA 1.18 (95% CI 0.95 to 1.46), for decreasing PA 1.13 (95% CI 0.89 to 1.45) and for increasing PA 1.02 (95% CI 0.84 to 1.23). The odds of injurious falls for those with low level of PA were 1.06 (95% CI 0.83 to 1.35), for some PA 1.12 (95% CI 0.91 to 1.39), for decreasing PA 1.37 (95% CI 1.08 to 1.73) and for increasing PA 1.06 (95% CI 0.87 to 1.28) (online supplemental figure 5). When adjusting for these covariates at survey 8, the odds of non-injurious falls for those with low level of PA were 1.31 (95% CI 1.04 to 1.65), for some PA 1.08 (95% CI 0.87 to 1.35), for decreasing PA 1.05 (95% CI 0.82 to 1.35), for increasing PA 1.02 (95% CI 0.84 to 1.24). The odds of injurious falls for low level of PA were 0.97 (95% CI 0.76 to 1.24), for some PA 1.07 (95% CI 0.87 to 1.32), for decreasing PA 1.19 (95% CI 0.94 to 1.50), for increasing PA 1.01 (95% CI 0.83 to 1.22) (online supplemental figure 5).

Sensitivity analyses showed similar associations between latent class memberships of PA participation and odds of subsequent non-injurious and injurious falls on the crude complete-case analysis when adjusted for ARIA+, BMI, the number of health conditions, education level and ability to manage income (online supplemental figures 6 and 7 for confounders collected at survey 2 and survey 8, respectively) and ARIA+, BMI, the number of health conditions and mean perceived stress (online supplemental figures 8 and 9 for confounders collected at survey 2 and survey 8, respectively).

## Discussion

We identified five distinct patterns of amounts of PA participation over 18 years: consistently lower level of PA (below 100 min/week PA at most surveys), consistently some PA close to and without reaching the lower limit of PA guideline, decreasing PA and maintaining ≥150 min/week, increasing PA and maintaining ≥150 min/week and consistently highly active and maintained ≥300 min/week of PA. The less active PA participation patterns were associated with increased odds of falls (injurious and non-injurious falls) at follow-up compared with the consistently highly active pattern. The increased odds of non-injurious falls for those with consistently lower levels of activity compared with those with consistently high levels remained after adjusting for confounders measured in survey 2 and survey 8; but the association was not found for injurious falls.

Our findings are similar to the previous study that reported a higher risk of falls among people with consistently lower levels of PA (adjusted rate ratio 1.28, 95% CI 1.14 to 1.44) and with decreasing PA participation pattern (adjusted incidence relative risk 1.14, 95% CI 1.01 to 1.28), compared with people with consistently high PA.^15^ While this previous study examined the associations between different PA participation patterns with the number of falls (ie, continuous variable) or any falling (ie, a binary outcome and dichotomised into yes/no about whether participants had experienced any falls regardless of injury)^15^ and did not examine the relationship with fall-related injuries. The findings in our study identify the different magnitudes and the distinct associations with non-injurious and injurious falls.

Some previous studies demonstrated a U-shaped relationship between PA and falls, with an increase in falls among those with low and high levels of PA.^29 30^ In our study, there was no evidence of an increase in subsequent non-injurious and injurious falls among those with increasing PA participation patterns. Previous associations between PA and falls were explored either when PA and falls were evaluated at the same time point^16^ or when PA was evaluated at one time point and falls were prospectively collected after a short follow-up period (ranging between 1 and 4.5 years).^16 29 30^ In contrast, our study explored the association between different patterns of PA amounts over an 18-year span and falls in later life when women were in their 70s. Our study underscores the importance of maintaining PA at least at the recommended level. Further, with no evidence to suggest an increase in fall risk with increases in PA from mid-life to older age, our findings support the promotion of PA for health benefits. Reviews have also demonstrated the benefits of PA on physical function,^31^ frailty^32^ and bone health,^33^ which are risk factors for injurious falls among older adults.

### Clinical implications

The findings of a higher risk of non-injurious falls among those who consistently had lower levels of PA underscores the urgent need for a lifelong commitment to PA participation, with at least 150 min/week, as recommended by WHO.^6^ Meanwhile, there is no evidence of an increased fall risk among women with other PA participation patterns, particularly those with patterns that had PA participation above WHO’s recommended levels from mid-life to older age. Individuals should be encouraged to adopt a lifelong commitment to the recommended level of PA for overall health benefits.^5 6^

### Strengths

The strength of the study included the use of large longitudinal PA data, in which women of similar age (ie, aged 45–50 years in 1996) were randomly recruited. Meanwhile, another strength of this study was the use of regular surveys every 3 years to capture the distinct long-term changing patterns of PA amounts from mid-life to older age over an 18-year span, which has not been explored widely in the literature. In addition, we prospectively assessed the associations between distinct patterns of amounts of PA participation and subsequent non-injurious and injurious falls 3 years later with the adjustment of potential covariates as identified from the DAG. The DAG was constructed based on the hypothesised theoretical framework, and we acknowledge that grouping of variables may introduce some levels of abstraction. However, the use of DAG reduces the potential source of bias such as overadjustment bias in an exposure-and-outcome relationship. This allows a precise estimate and improves the ability to draw conclusions.

### Limitations and future research

Nevertheless, several limitations deserve further discussion. First, latent class memberships of amounts of PA from the latent class analysis depend on variation within the data. There are uncertainties of class assignment in latent class analysis as each participant was assigned to the class membership to which membership probability was the largest.^34^ However, amounts of PA participation patterns identified in our study were similar to the PA participation patterns previously described in a systematic review of cohort studies in other countries, including the UK, the USA and Taiwan.^11^ Participants were asked to report the amount of PA they performed in the past week, which may increase the risk of biases (eg, misclassification bias). However, the use of repeated measures on PA may have mitigated this risk of bias. Recall of PA in the past week has also shown acceptable psychometric properties.^35^ In addition, we categorised self-reported PA according to the guideline’s recommended levels of weekly PA.^6^ Although we consider this method to enable interpretability and practical relevance in understanding the patterns of amounts of PA participation from mid-life to older age, future research could further investigate the patterns of PA participation by using device-measured PA.

Furthermore, the ALSWH has been designed to collect a range of health data longitudinally since 1996 using standardised questions, and the exact type of PA, the number of falls or severity of the injurious falls (eg, fall-related mortality) were not collected. Given the existing evidence that balance and strength exercises conducted in trials reduce falls in older adults,^7^ future studies should also consider the associations between patterns of types of PA participation and falls. Future research could also use linked administrative data to examine the association between different PA participation patterns and number of falls that require medical attention or fall-related mortality. Another limitation is that our study focused on women in Australia so replication of the identified trajectories in other cohorts such as Australian men is needed. Inevitably, a total of 3981 (33.7%) individuals were excluded from the analyses due to missing responses about falls. Individuals who were excluded had a higher proportion of no formal education and had difficulty in managing on their income, compared with those included in the analysis (online supplemental table 3). Hence, this may pose selection bias and limit the generalisability of our findings to people with lower socioeconomic conditions, which warrant further investigation. Finally, with the loss of significant association between patterns of consistently some PA, decreasing PA and falls after adjusting for potential confounders, future studies could explore the influence of these factors (ie, ARIA+, health condition, education, ability to manage income and perceived stress) on the association of patterns of PA and falls.

## Conclusion

We identified five distinct patterns of amounts of PA participation from mid-life to older age (ie, consistently lower level of PA, consistently some PA, decreasing PA, increasing PA and consistently highly active) among Australian women. Compared with those who were consistently highly active, a higher odd of injurious and non-injurious falls were found among those with low or decreasing PA. After adjusting for confounders, there remained an increased odds of non-injurious falls for those with consistently lower levels of PA. Results from this study support ongoing PA participation from mid-life to older age for falls prevention.

## Data Availability

Data may be obtained from a third party and are not publicly available. The data are not publicly available due to the data sharing agreement with the Australian Longitudinal Study on Women’s Health (ALSWH) However, data can be accessed when researchers request the ALSWH data at https://alswh.org.au/for-data-users/applying-for-data/quick-guide/.
