# Supplementary material for: Falls and patterns of physical activity participation over 18 years in the Australian Longitudinal Study on Women’s Health
Source: Br J Sports Med. 2024 Jul 24;58(16):919–29. doi: 10.1136/bjsports-2024-108262 (PMC11347968; doi:10.1136/bjsports-2024-108262)
Supplement: Supplementary data [file bjsports-2024-108262supp001.pdf]

Appendices

Supplementary Table 1 Details of the variables collected in the ALSWH and constructed in the directed acyclic graph (DAG)

Supplementary Table 2 Guidelines for Reporting on Latent Trajectory Studies (GRoLTS) Checklist

Supplementary Table 3 Characteristics between samples included in the multinominal logistic regression versus those excluded due to missing outcome in survey 9 in the 1946-51 born cohort in the Australian Longitudinal Study on Women's Health

Supplementary Table 4 Distribution of physical activity participation

Supplementary Table 5 Goodness of fit statistics for 2- to 6-cluster models

Supplementary Table 6 2x2 table for 5-cluster memberships assigned from repeated-measures latent class analysis when the sample size of n=11,796 and n=7,815 were used

Supplementary Figure 1 Directed acyclic graph showing the hypothesised association between ongoing physical activity participation (exposure) and injurious falls (outcome)

Supplementary Figure 2 Line graph of fit statistics based on the repeated measures latent class analysis of physical activity over time

Supplementary Figure 3 Proportion of women reported non-injurious and injurious falls from survey 4 (2004) to survey 9 (2019) (n=11,796).

Supplementary Figure 4 Associations between the different subgroups of physical activity participation pattern and subsequent falls and injurious falls from crude analysis

Supplementary Figure 5 Associations between the different subgroups of physical activity participation pattern and subsequent falls and injurious falls when adjusted for ARIA+, body mass index, number of health condition and mean perceived stress in survey 2 and survey 8

Supplementary Figure 6 Complete case analysis for the associations between physical activity participation patterns and subsequent falls and injurious falls when adjusted for survey 2 ARIA+, body mass index, number of health conditions, education and ability to manage on income (n=7,062)

Supplementary Figure 7 Complete case analysis for the associations between physical activity participation patterns and subsequent falls and injurious falls when adjusted for survey 8 ARIA+, body mass index, number of health conditions, education and ability to manage on income (n=7,140)

Supplementary Figure 8 Complete case analysis for the associations between physical activity participation patterns and subsequent falls and injurious falls when adjusted for survey 2 ARIA+, body mass index, number of health conditions, perceived stress (n=7,128)

Supplementary Figure 9 Complete case analysis for the associations between physical activity participation patterns and subsequent falls and injurious falls when adjusted for survey 8 ARIA+, body mass index, number of health conditions, perceived stress (n=7,174)

Kwok WS, *et al. Br J Sports Med* 2024; 58:919–929. doi: 10.1136/bjsports-2024-108262

Supplementary Table 1 Details of the variables collected in ALSWH and constructed in the directed acyclic graph (DAG)

**Concepts / Node in the DAG**	**Variables included within the concepts**	**Information on how the data were collected in the survey**
Physical activity (Exposure) [(1)]	Walking	How many times did you walk briskly (for recreation or exercise, or to get from place to place) activity last week? Only count the number of times when the activity lasted for 10 minutes or more.<br><br>If you add up all the times you spent in each activity last week, how much time did you spend altogether in walking briskly (for recreation or exercise, or to get from place to place)?
	Moderate leisure activity	How many times did you do moderate leisure activity (like social tennis, moderate exercise classes, recreational swimming, dancing) last week? Only count the number of times when the activity lasted for 10 minutes or more.<br><br>If you add up all the times you spent in each activity last week, how much time did you spend altogether doing moderate leisure activity (like social tennis, moderate exercise classes, recreational swimming, dancing)?
	Vigorous leisure activity	How many times did you do vigorous leisure activity (that makes you breathe harder or puff and pant like aerobics, competitive sport, vigorous cycling, running, swimming) last week? Only count the number of times when the activity lasted for 10 minutes or more.<br><br>If you add up all the times you spent in each activity last week, how much time did you spend altogether doing vigorous leisure activity (that makes you breathe harder or puff and pant like aerobics, competitive sport, vigorous cycling, running, swimming)
Falls (Outcome)	Falls without injury (non-injurious falls), falls with injury (injurious falls)	"In the last twelve months have you 1) had a fall to the ground; 2) been injured as a result of a fall; 3) needed to seek medical attention for an injury from a fall<br>[Non-injurious falls, response 'Yes' to the statement 1) had a fall to the ground and 'No' to statements 2) been injured as a result of a fall; and 3) needed to seek medical attention for an injury from a fall]<br>[Injurious falls, response 'Yes' to the statements 2) been injured as a result of a fall; 3) needed to seek medical attention for an injury from a fall
Body mass index	Body mass index	Based on self-reported weight and height
Osteoporosis	Osteoporosis	In the past three years, have you been diagnosed with or treated for osteoporosis?<br>[Response if 'Yes, in the past 3 years']
Cognition and Mood	Memory	In the last 12 months, have you had poor memory?<br>[Responses: "Never" or "Rarely" or "Sometimes" or "Often"]

	Short form (SF36) Mental health [(2)]	For each question, please give the one answer that comes closest to the way you have been feeling. How much of the time during the past 4 weeks?<br>• Have you been a very nervous person<br>• Have you felt so down in the dumps that nothing could cheer you up<br>• Have you felt calm and peaceful<br>• Have you felt down<br>• Have you been a happy person<br>[Response to the above statement: “All of the time” or “Most of the time” or “A good bit of the time” or “some of the time” or “A little of the time” or “None of the time”]
Mobility and Balance	Loss of balance	In the last 12 months, have you had dizziness, loss of balance? [Responses: “Never” or “Rarely” or “Sometimes” or “Often”]
	SF36 Physical functioning [(2)]	The following questions are about activities you might do during a typical day. Does your health now limit you in these activities? If so, how much?<br>• Vigorous activities, such as running, lifting heavy objects, participating in strenuous sports<br>• Moderate activities, such as moving a table, pushing a vacuum cleaner, bowling or playing golf<br>• Lifting or carrying groceries<br>• Climbing 3 flights of stairs<br>• Climbing one flight of stairs<br>• Bending, kneeling or stooping<br>• Walking more than one kilometre<br>• Walking half a kilometre<br>• Walking 100 metres<br>• Bathing or dressing yourself<br>[Response to the above statement: “Yes, limited a lot”, “Yes, limited a little”, “No, not limited at all”
Environment	Accessibility Remoteness Index of Australia scale (ARIA+) [(3)]	Accessed according to the remoteness of geographic classification and classified as “Major cities of Australia” or “Inner regional Australia” or “Outer regional Australia” or “Remote Australia” or “Very remote Australia”
Socioeconomic status	Education[a]	What is the highest qualification you have completed? [Response: “No formal education” or “School Certificate” or “Higher school certificate” or “Trade/Apprentice // Certificate/ Diploma” or “University degree // Higher degree”
	Manage on income[(4)]	How do you manage on the income you have available? [Response: “It is impossible” or “It is difficult all the time” or “It is difficult some of the time” or “It is not too bad” or “It is easy”]
Health conditions	Number of health conditions	In the past three years, have you been diagnosed with or treated for<br>• Diabetes<br>• Impaired glucose tolerance<br>• Osteoarthritis<br>• Rheumatoid arthritis<br>• Heart disease (including heart attack, angina0<br>• Thrombosis (a blood clot)<br>• Hypertension (high blood pressure)<br>• Stroke

		• Parkinson's disease<br>• Mild cognitive impairment<br>Alzheimer's disease or dementia<br>• Low iron level (iron deficiency or anaemia)<br>• Asthma<br>• Bronchitis/ emphysema<br>• Breast cancer<br>• Cervical cancer<br>• Lung cancer<br>• Bowel cancer (colorectal cancer)<br>• Skin cancer (including melanoma)<br>• Other cancer (please specify)<br>• Depression<br>• Anxiety / nervous disorder<br>• Other Psychiatric disorder<br>• Chronic fatigue syndrome
Pain	SF 36 Bodily pain [(2)]	How much BODILY pain have you had during the PAST 4 WEEKS?<br>"None", "Very mild", "Mild", "Moderate", "Severe", "very Severe"<br>During the PAST FOUR WEEKS, how much did PAIN interfere with your normal work (including both work outside the home and housework)?<br>"Not at all", "A little bit", "Moderately", "Quite a bit". Extremely"
Polypharmacy[b]		In the past four weeks, have you taken<br>• Any medications prescribed by the doctor?<br>In the past four weeks, have you taken<br>• Aspirin<br>• Paracetamol<br>• Ibuprofen
Participation	Require help	Do you regularly need help with daily tasks because of long-term illness, disability or frailty? (eg personal care, getting around, preparing meals etc)?<br>[Response: "Yes" or "No"]
	Taking care of grandchildren or other people's children	Do you regularly provide (unpaid) care for grandchildren or other people's children?<br>[Response: "Yes, daily" or "Yes, weekly" or "Yes, occasionally" or "No, never"
	Volunteer work	Do you do any volunteer work for any community or social organisations (eg fundraising, community welfare, church activities, organising groups or classes, etc)?<br>[Response: "Not at all", or "every day" or "every week" or "every month" or "less than once a month"]
	Provide help to others	Do you regularly provide care or assistance (eg personal care, transport) to any other person because of their long-term illness, disability or frailty?<br>• for someone who lives with you<br>• for someone who lives elsewhere<br>[Response to the above statement: "Yes" or "No"]

	SF36 Social functioning [(2)]	During the past 4 weeks, to what extent has your physical health or emotional problems interfered with your normal social activities with family, friends, neighbours or groups?<br>[Responses can be: "Not at all", " A little bit", "Moderately", "Quite a bit", "Extremely"<br><br>During the past 4 weeks, how much of the time has your physical health or emotional problems interfered with your social activities (like visiting friends, relatives, etc)?<br>[Response to the above statement: "All of the time" or "Most of the time" or "A good bit of the time" or "some of the time" or "A little of the time" or "None of the time"]
	SF36 Role - Physical [(2)]	During the past 4 weeks, have you had any of the following problems with your work (including your work outside the home and housework) or other regular daily activities as a result of your physical health?<br>• Cut down on the amount of time you spent on work or other activities<br>• Accomplished less than you would like<br>• Were limited in the kind of work or other activities<br>• Had difficulty performing the work or other activities (for example it took extra effort)<br>[Response to the above statement: "Yes" or "No"]
	SF36 Role - Emotional [(2)]	During the past 4 weeks, have you had any of the following problems with your work or other regular daily activities as a result of any emotional problems (such as feeling depressed or anxious)?<br>• Cut down on the amount of time you spent on work or other activities<br>• Accomplished less than you would like<br>• Didn't do work or other activities as carefully as usual<br>[Response to the above statement: "Yes" or "No"]
Stress	Perceived stress [(5)]	The scale assessed items in specific life domains including:<br>• Own health<br>• Health of other family members<br>• Work/employment<br>• Living arrangements<br>• Study<br>• Money<br>• Relationship with parents<br>• Relationship with partner/ spouse<br>• Relationship with other family members<br><br>[Response to the above: Not applicable, not at all stressed, somewhat stressed, moderately stressed, very stressed, extremely stressed;]<br>The possible mean score ranges from 0 to 4, with 0 refers to Not applicable/ not at all stressed, 1 refers to somewhat stressed, 2 refers to moderately stressed, 3 refers to very stressed and 4 refers to extremely stressed
Vision impairment	Vision impairment	In the past three years, have you been diagnosed with or treater for<br>• Macular degeneration<br>• Cataracts<br>• Glaucoma

[a]Highest qualification completed was not collected in survey 9 and therefore data last collected in survey 6 (in 2010) was used. If participants had missing data of education in survey 6, the data of education collected in survey 1 (in 1996) was used.

[b] Questions listed are only based on consistent questions about medications asked from survey 2 to survey 9. Among survey 2 (1998) to survey 4 (2004), participants also reported any medications taken for various conditions for: nerves/anxiety/worries, stress (difficult coping), to help you sleep, tiredness/fatigue, depression, menopausal symptoms, high blood pressure, high cholesterol, heart problems, arthritis, diabetes or blood sugar, asthma, digestive/bowel problems, skin problems, headache, backache, other pain, any other chronic (long-term) illness or condition. In survey 5 (2007) and survey 6 (2010), participants reported all the medications in an open-ended question (i.e., writing down the names of all the medications).

Kwok WS, *et al. Br J Sports Med* 2024; 58:919–929. doi: 10.1136/bjsports-2024-108262

Supplementary Table 2 Guidelines for Reporting on Latent Trajectory Studies (GRoLTS) Checklist[(6)]

Checklist item	Reported
1. Is the metric of time used in the statistical model reported	Yes
2. Is information presented about the mean and variance of time within a wave?	No, the data was collected every three years (time structured)
3a. Is the missing data mechanism reported?	Yes
3b. Is a description provided of what variables are related to attrition/missing data?	Yes
3c. Is a description provided of how missing data in the analyses were dealt with?	Yes
4. Is information about the distribution of the observed variables included?	Yes
5. Is the software mentioned?	Yes
6a. Are alternative specifications of within-class heterogeneity considered (e.g., LGCA vs. LGMM) and clearly documented? If not, was sufficient justification provided as to eliminate certain specifications from consideration?	Not applicable as the cohort was similar with age
6b. Are alternative specifications of the between-class differences in variance–covariance matrix structure considered and clearly documented? If not, was sufficient justification provided as to eliminate certain specifications from consideration?	Not applicable
7. Are alternative shape/functional forms of the trajectories described?	Yes
8. If covariates have been used, can analyses still be replicated?	Yes
9. Is information reported about the number of random start values and final iterations included?	Yes
10. Are the model comparison (and selection) tools described from a statistical perspective?	Yes
11. Are the total number of fitted models reported, including a one-class solution?	Yes
12. Are the number of cases per class reported for each model (absolute sample size, or proportion)?	Yes
13. If classification of cases in a trajectory is the goal, is entropy reported?	Yes
14a. Is a plot included with the estimated mean trajectories of the final solution?	Yes, Figure 3
14b. Are plots included with the estimated mean trajectories for each model?	Yes, model fit statistics have been provided in Supplementary Table 5
14c. Is a plot included of the combination of estimated means of the final model and the observed individual trajectories split out for each latent class?	Yes
15. Are characteristics of the final class solution numerically described (i.e., means, SD/SE, n, CI, etc.)?	Yes
16. Are the syntax files available (either in the appendix, supplementary materials, or from the authors)?	Yes, available from authors

Supplementary Table 3 Characteristics between samples included in the multinominal logistic regression versus those excluded due to missing outcome in survey 9 in the 1946-51 born cohort in the Australian Longitudinal Study on Women's Health

	**Total**		**Consistently low level of PA**		**Consistently some PA**		**Decreasing PA**		**Increasing PA**		**Consistently highly active**	
**Characteristics**	Participants included n=7,815	Participants excluded n=3,981	Participants included n=1,087	Participants excluded n=1,056	Participants included n=1,482	Participants excluded n=665	Participants included n=956	Participants excluded n=389	Participants included n=2,465	Participants excluded n=1,075	Participants included n=1,825	Participants excluded n=796
**Age, years**	49.5 (1.5)	49.6 (1.5)	49.5 (1.4)	49.5 (1.5)	49.4 (1.5)	49.6 (1.5)	49.5 (1.5)	49.6 (1.4)	49.4 (1.5)	49.5 (1.5)	49.5 (1.4)	49.6 (1.5)
**Smoking status**												
Never smoker	4,296 (55)	1,810 (45)	530 (49)	428 (41)	857 (58)	323 (49)	471 (49)	166 (43)	1,410 (57)	535 (50)	1,028 (56)	358 (45)
Ex-smoker	2,002 (26)	832 (21)	243 (22)	210 (20)	333 (22)	123 (18)	289 (30)	100 (26)	642 (26)	229 (21)	495 (27)	170 (21)
Smoker	978 (13)	692 (17)	225 (21)	236 (22)	182 (12)	84 (13)	131 (14)	74 (19)	250 (10)	167 (16)	190 (10)	131 (16)
Missing	539 (7)	647 (16)	89 (8)	182 (17)	110 (7)	135 (20)	65 (7)	49 (13)	163 (7)	144 (13)	112 (6)	137 (17)
**Menopausal Status**[a]												
Pre-menopausal	1,875 (24)	694 (17)	217 (20)	169 (16)	344 (23)	124 (19)	208 (22)	68 (17)	623 (25)	185 (17)	483 (26)	148 (19)
Peri-menopausal	1,919 (25)	830 (21)	226 (21)	208 (20)	365 (25)	129 (19)	217 (23)	83 (21)	630 (26)	253 (24)	481 (26)	157 (20)
Post-menopausal/ Surgical menopause	2,571 (33)	1,558 (39)	421 (39)	455 (43)	500 (34)	254 (38)	343 (36)	154 (40)	744 (30)	399 (37)	563 (31)	296 (37)
HRT use or OCP use	1,160 (15)	509 (13)	169 (16)	118 (11)	217 (15)	83 (12)	149 (16)	49 (13)	389 (16)	157 (15)	236 (13)	102 (13)
Missing	290 (4)	390 (10)	54 (5)	106 (10)	56 (4)	75 (11)	39 (4)	35 (9)	79 (3)	81 (8)	62 (3)	93 (12)
**Education, n (%)**												
No formal education	1,017 (13)	967 (24)	262 (24)	358 (34)	202 (14)	168 (25)	154 (16)	91 (23)	233 (9)	213 (20)	166 (9)	137 (17)
School or intermediate certificate or higher school or leaving certificate	5,361 (69)	2,626 (66)	715 (66)	645 (61)	1,004 (68)	430 (65)	674 (71)	259 (67)	1,696 (69)	721 (67)	1,272 (70)	571 (72)
University degrees of above	1,382 (18)	343 (9)	97 (9)	45 (4)	267 (18)	60 (9)	122 (13)	33 (8)	523 (21)	126 (12)	373 (20)	79 (10)
Missing	55 (1)	45 (1)	13 (1)	*	*	*	*	*	13 (1)	15 (1)	14 (1)	*
**Ability to manage on income, n (%)**												
Impossible	120 (2)	86 (2)	40 (4)	33 (3)	25 (2)	14 (2)	18 (2)	*	29 (1)	19 (2)	*	13 (2)
Difficult always	805 (10)	518 (13)	166 (15)	184 (17)	150 (10)	72 (11)	116 (12)	52 (13)	244 (10)	125 (12)	129 (7)	85 (11)
Difficult sometimes	2,036 (26)	1,031 (26)	315 (29)	264 (25)	439 (30)	178 (27)	265 (28)	110 (28)	603 (24)	280 (26)	414 (23)	199 (25)
Not too bad	3,068 (39)	1,272 (32)	366 (34)	298 (28)	570 (38)	208 (31)	348 (36)	133 (34)	1,020 (41)	372 (35)	764 (42)	261 (33)
Easy	1,241 (16)	391 (10)	107 (10)	87 (8)	189 (13)	54 (8)	139 (15)	35 (9)	409 (17)	123 (11)	397 (22)	92 (12)
Missing	545 (7)	683 (17)	93 (9)	190 (18)	109 (7)	139 (21)	70 (7)	52 (13)	160 (6)	156 (15)	113 (6)	146 (18)

**Location (ARIA+), n (%)**												
Major cities	2,596 (33)	1,342 (34)	313 (29)	339 (32)	487 (33)	225 (34)	293 (31)	122 (31)	879 (36)	406 (38)	624 (34)	250 (31)
Inner regional	3,227 (41)	1,543 (39)	455 (42)	397 (38)	624 (42)	248 (37)	387 (40)	154 (40)	975 (40)	396 (37)	786 (43)	348 (44)
Outer regional, remote and very remote	1,991 (25)	1,096 (28)	319 (29)	320 (30)	371 (25)	192 (29)	276 (29)	113 (29)	611 (25)	273 (25)	414 (23)	198 (25)
Missing	*	*	*	*	*	*	*	*	*	*	*	*
**Body mass index, n (%)**												
< 18.5 kg/m^2	94 (1)	52 (1)	12 (1)	*	15 (1)	*	*	*	29 (1)	14 (1)	29 (2)	18 (2)
≥18.5 to <25 kg/m^2	3,696 (47)	1,551 (39)	322 (30)	326 (31)	616 (42)	222 (33)	407 (43)	168 (43)	1,277 (52)	473 (44)	1,074 (59)	362 (45)
≥25 to <25 kg/m^2	2,334 (30)	1,096 (28)	343 (32)	277 (26)	460 (31)	180 (27)	312 (33)	108 (28)	748 (30)	310 (29)	471 (26)	221 (28)
≥30 kg/m^2	1,369 (18)	842 (21)	336 (31)	309 (29)	325 (22)	169 (25)	183 (19)	77 (20)	325 (13)	179 (17)	200 (11)	108 (14)
Missing	322 (4)	440 (11)	74 (7)	135 (13)	66 (4)	87 (13)	45 (5)	32 (8)	86 (3)	99 (9)	51 (3)	87 (11)
**Number of health conditions, n (%)**												
Nil	2,667 (34)	1,267 (32)	332 (31)	302 (29)	450 (30)	200 (30)	312 (33)	135 (35)	885 (36)	356 (33)	688 (38)	274 (34)
1	2,421 (31)	1,080 (27)	300 (28)	279 (26)	450 (30)	179 (27)	290 (30)	93 (24)	799 (32)	314 (29)	582 (32)	215 (27)
2	1,429 (18)	650 (16)	217 (20)	193 (18)	311 (21)	102 (15)	186 (19)	64 (16)	407 (17)	169 (16)	308 (17)	122 (15)
≥3	1,031 (13)	620 (15)	187 (16)	186 (18)	220 (16)	113 (17)	131 (14)	67 (17)	301 (12)	156 (15)	192 (10)	98 (13)
Missing	267 (3)	364 (9)	51 (5)	96 (9)	51 (3)	71 (11)	37 (4)	30 (8)	73 (3)	80 (7)	55 (3)	87 (11)
**Perceived stress, score, mean (SD)[a]**	0.61 (0.48)	0.63 (0.50)	0.69 (0.54)	0.69 (0.56)	0.64 (0.49)	0.64 (0.53)	0.62 (0.49)	0.63 (0.54)	0.59 (0.46)	0.59 (0.51)	0.54 (0.45)	0.60 (0.52)
**SF 36 Physical Function[b]**	86 (16)	81 (21)	78 (21)	73 (26)	84 (17)	81 (19)	85 (16)	83 (20)	88 (14)	85 (18)	91 (12)	87 (17)
**SF 36 Mental Health[c]**	75 (17)	72 (20)	70 (20)	67 (21)	73 (18)	72 (19)	74 (17)	72 (20)	76 (17)	73 (19)	78 (15)	75 (19)

Participants characteristics at survey 2 (1998) between participants who had responded falls outcome in survey 9 (i.e., included in the multinominal logistic regression) versus those with missing outcome in survey 9 in the 1946-51 born cohort in the Australian Longitudinal Study on Women's Health.
ARIA+ = Accessibility Remoteness Index of Australia scale; PA=physical activity
For participants confidentiality, missing data with a small sample size (n<10) is presented with * .
Percentage may not add up to 100 due to rounding
[a]Mean perceived stress score measured with the ALSWH perceived stress scale and was presented according to the data available. Sample size included from left to right column n=7,293; n=3,329; n=1,001; n=874; n=1,377; n=531; n=890; n=339; n=2,312; n=929; n=1,713; n=656.
[b] SF36 Physical Function was presented according to the data available Sample size included from left to right column n=7513; n=3583; n=1,032; n=950; n=1,425; n=591; n=914; n=356; n=2,383; n=984; n=1,759; n=702
[c]SF36 Mental Health was presented according to the data available Sample size included from left to right column n=7,533; n=3,598; n=1,034; n=958; n=1,428; n=592; n=918; n=358; n=2,387; n=990; n=1,766; n=700;

Supplementary Table 4 Distribution of physical activity participation

	Survey 2, 1998	Survey 3, 2001	Survey 4, 2004	Survey 5, 2007	Survey 6, 2010	Survey 7, 2013	Survey 8, 2016
**0 min**	1,886 (16)	1,824 (15)	1,731 (15)	1,694 (14)	1,704 (14)	1,520 (13)	1,456 (12)
**1 to <150 min**	2,724 (23)	3,185 (27)	2,265 (19)	2,000 (17)	1,946 (17)	1,693 (14)	1,552 (13)
**150 to <300 min**	2,549 (22)	2,373 (20)	2,280 (19)	2,216 (19)	2,002 (17)	1,749 (15)	1,668 (14)
**≥ 300 min**	3,318 (28)	2,890 (25)	3,666 (31)	3,968 (34)	3,718 (32)	3,756 (32)	3,650 (31)
**Missing response**	1,319 (11)	1,524 (13)	1,854 (16)	1,918 (16)	2,426 (21)	3,078 (26)	3,470 (29)

Distribution of physical activity participation included in the latent class analysis (n=11,796).
Data was presented in n (%)

Supplementary Table 5 Goodness of fit statistics for 2- to 6-cluster models

	1-cluster model	2-cluster model	3-cluster model	4-cluster model	5-cluster model	6-cluster model
**Log likelihood**	-89578.22	-84314.87	-82910.40	-82499.39	-82321.23	-82200.21
**AIC**	26261.34	15778.65	13013.71	12235.68	11923.37	11725.34
**BIC**	26416.23	16095.79	13493.12	12877.35	12727.31	12691.53
**Degrees of freedom**	16362	16340	16318	16296	16274	16252
**G square**	26219.34	15692.65	12883.72	12061.68	11705.37	11463.34
**Entropy**	1.00	0.67	0.63	0.6	0.55	0.52
**Cluster membership, n (%)**	1. 11,796 (100)	1. 6,071 (51)<br>2. 5,725 (49)	1. 4,473 (38)<br>2. 4,599 (39)<br>3. 2,724 (23)	1. 1,798 (15)<br>2. 4,993 (42)<br>3. 2,391 (20)<br>4. 2,614 (22)	1. 2,143 (18)<br>2. 2,174 (18)<br>3. 1,345 (11)<br>4. 3,540 (30)<br>5. 2,621 (22)	1. 2,450 (21)<br>2. 1,791 (15)<br>3. 2,162 (18)<br>4. 2,492 (21)<br>5. 1,336 (11)<br>6. 1,565 (13)

Random seeds=100000 and rho=1 was used in the different cluster models using repeated measures latent class analysis.
AIC: Akaike information criterion; BIC: Bayesian information criterion

Supplementary Table 6 2x2 table for 5-cluster memberships assigned from repeated-measures latent class analysis when the sample size of n=11,796 and n=7,815 were used

		Cluster membership assigned when sample size of n=7,815 was used					Participants with missing outcome in survey 9 and not included in repeated-measure latent class analysis when sample size of n=7,815 was used
Cluster membership assigned when sample size of n=11,796 was used	n	Consistently lower level of PA	Consistently some PA	Decreasing PA	Increasing PA	Consistently highly active	
	Consistently lower level of PA	1,079	3	0	5	0	1,056
	Consistently some PA	74	1,379	22	7	0	665
	Decreasing PA	105	8	770	34	39	389
	Increasing PA	1	130	512	1,822	0	1,075
	Consistently highly active	0	0	32	98	1,695	796
PA: physical activity							

Supplementary Figure 1 Directed acyclic graph showing the hypothesised association between ongoing physical activity participation (exposure) and injurious falls (outcome)

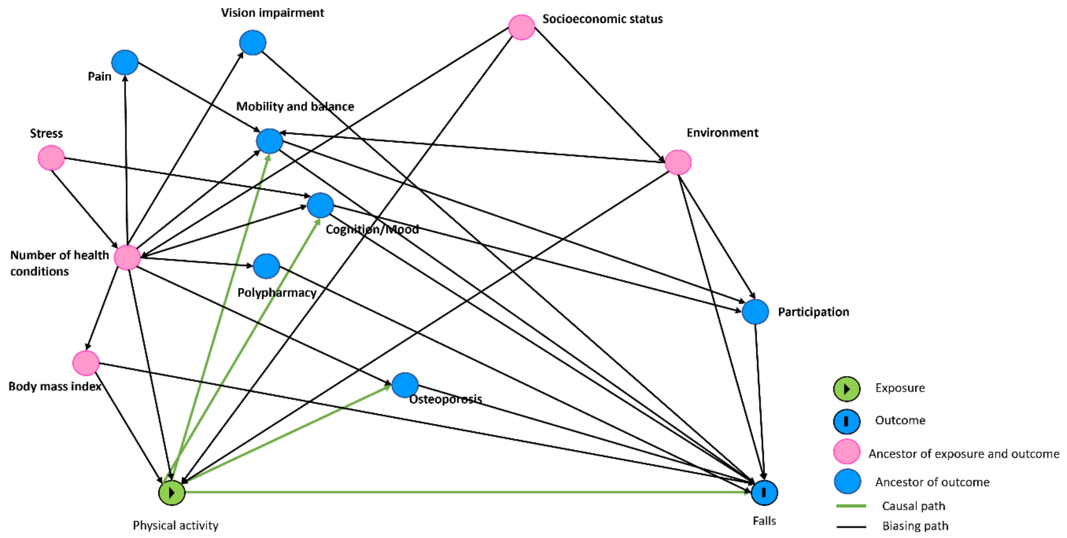

Kwok WS, *et al. Br J Sports Med* 2024; 58:919–929. doi: 10.1136/bjsports-2024-108262

Supplementary Figure 2 Line graph of fit statistics based on the repeated measures latent class analysis of physical activity over time

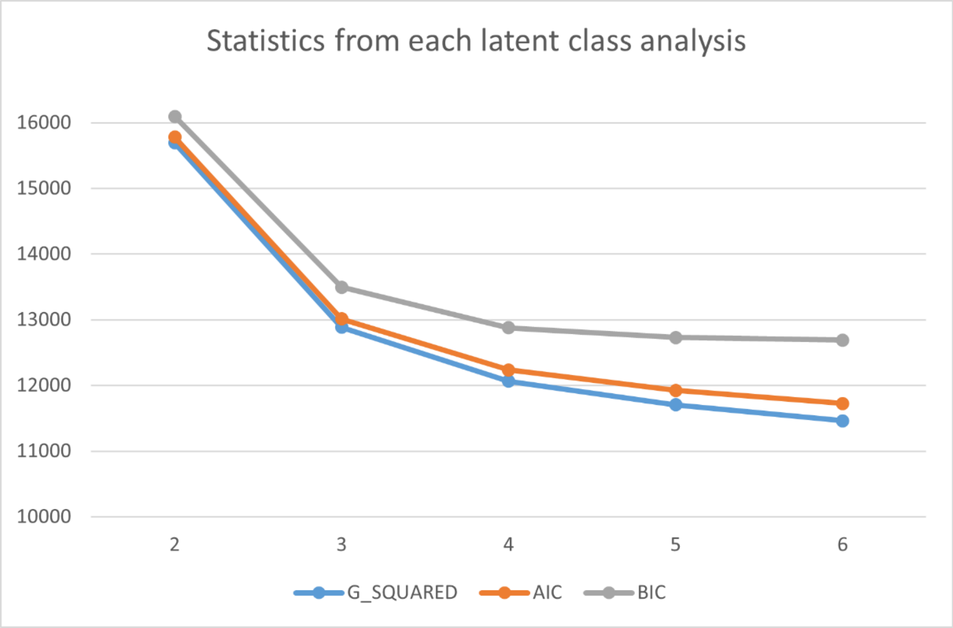

AIC: Akaike's information criterion; BIC: Bayesian information criterion

Kwok WS, *et al. Br J Sports Med* 2024; 58:919–929. doi: 10.1136/bjsports-2024-108262

Supplementary Figure 3 Proportion of women reported non-injurious and injurious falls from survey 4 (2004) to survey 9 (2019) (n=11,796).

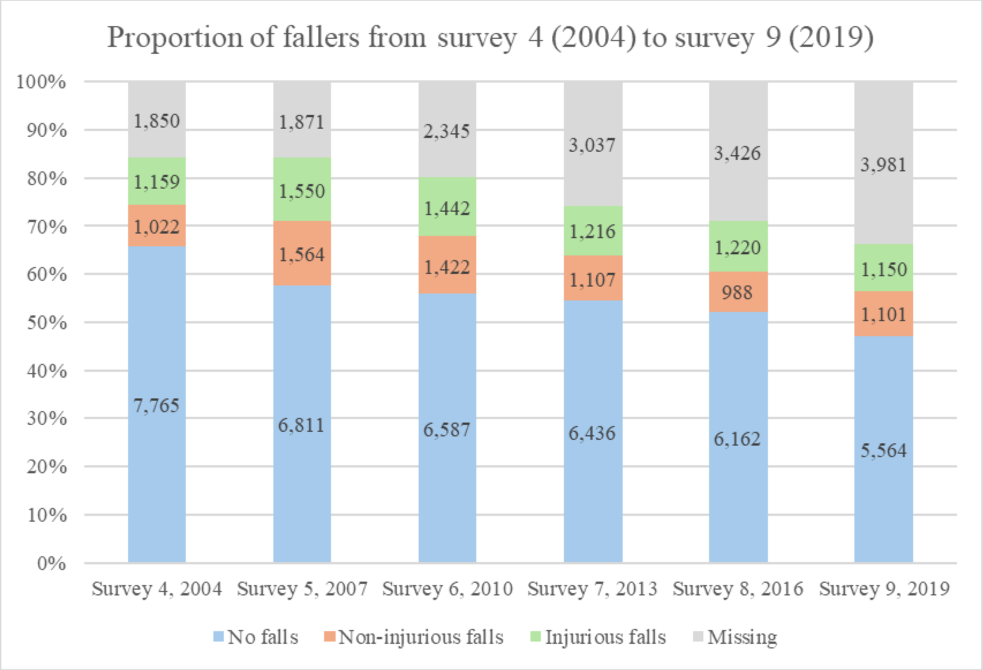

The number presented in the bar chart refers to the sample size (n).

Kwok WS, *et al. Br J Sports Med* 2024; 58:919–929. doi: 10.1136/bjsports-2024-108262

Supplementary Figure 4 Associations between the different subgroups of physical activity participation pattern and subsequent falls and injurious falls from crude analysis

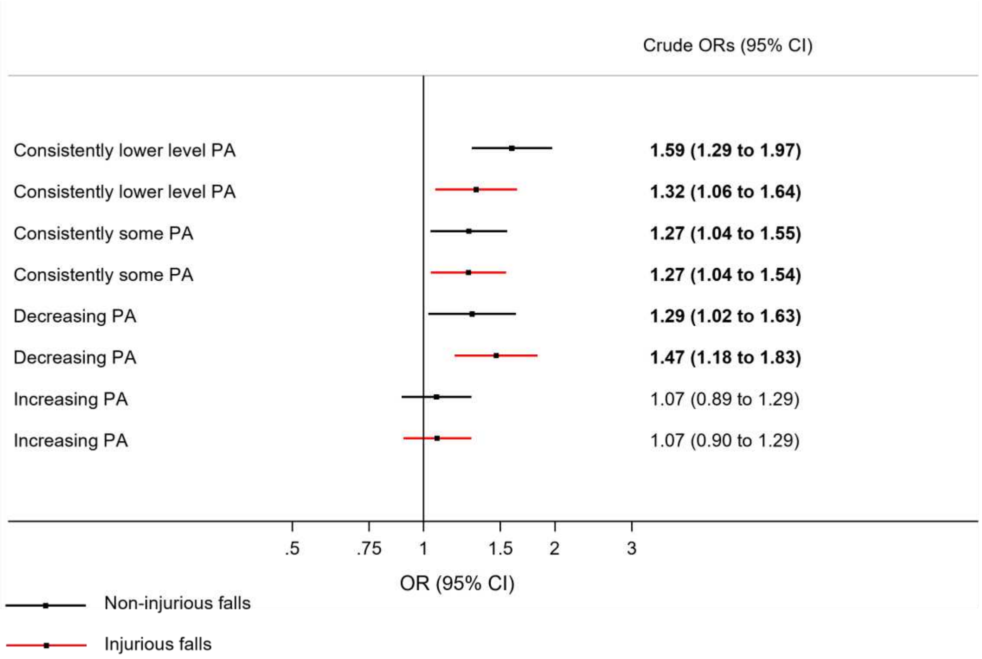

Association between different subgroups of physical activity participation pattern from middle age (survey 2, 1998, aged 47-52 years) to older age (survey 8, 2016, aged 65-70 years) and subsequent falls and injurious falls (aged 68-73 years) using multinomial logistic regression and presented in odds ratio and 95% confidence intervals (95% CI). Consistently highly active was used as the reference group. Crude model (n=7,815).

Kwok WS, *et al. Br J Sports Med* 2024; 58:919–929. doi: 10.1136/bjsports-2024-108262

Supplementary Figure 5 Associations between the different subgroups of physical activity participation pattern and subsequent falls and injurious falls when adjusted for ARIA+, body mass index, number of health condition and mean perceived stress in survey 2 and survey 8

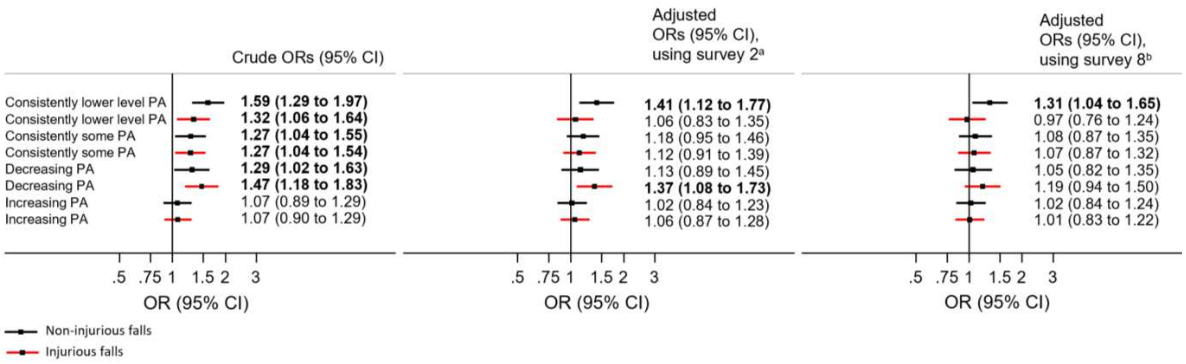

Association between the different subgroups of physical activity participation pattern from middle age (survey 2, 1998, aged 47-52 years) to older age (survey 8, 2016, aged 65-70 years) and subsequent falls and injurious falls (aged 68-73 years) using multinominal logistic regression and presented in odds ratio and 95% confidence intervals (95% CI). Crude model (n=7,815).

[a] adjusted for covariates (Accessibility Remoteness Index of Australia scale (ARIA+), body mass index, number of health condition and mean perceived stress) collected in survey 2 (n=7,128).

[b] adjusted for covariates (Accessibility Remoteness Index of Australia scale (ARIA+), body mass index, number of health condition and mean perceived stress) collected in survey 8 (n=7,174)

Kwok WS, *et al. Br J Sports Med* 2024; 58:919–929. doi: 10.1136/bjsports-2024-108262

Supplementary Figure 6 Complete case analysis for the associations between physical activity participation patterns and subsequent falls and injurious falls when adjusted for survey 2 ARIA+, body mass index, number of health conditions, education and ability to manage on income (n=7,062)

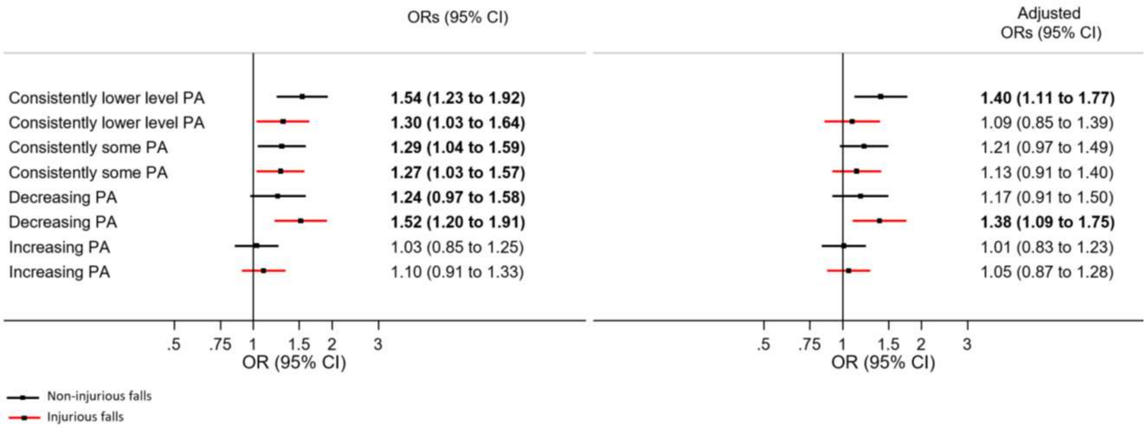

Complete case analysis (n=7,062) to examine the association between the different physical activity participation patterns from middle age (survey 2, 1998, aged 47-52 years) to older age (survey 8, 2016, aged 65-70 years) and subsequent falls and injurious falls (aged 68-73 years) using multinomial logistic regression and presented in odds ratio and 95% confidence intervals (95% CI). We classified complete case as for sample size only included participants who had been assigned with a physical activity participation pattern from latent class analysis, and responses in survey 9 falls, and all the covariates (Accessibility Remoteness Index of Australia scale (ARIA+), body mass index, number of health conditions, education and ability to manage on income) in survey 2. Adjusted odds ratio was adjusted for covariates collected in survey 2.

Supplementary Figure 7 Complete case analysis for the associations between physical activity participation patterns and subsequent falls and injurious falls when adjusted for survey 8 ARIA+, body mass index, number of health conditions, education and ability to manage on income (n=7,140)

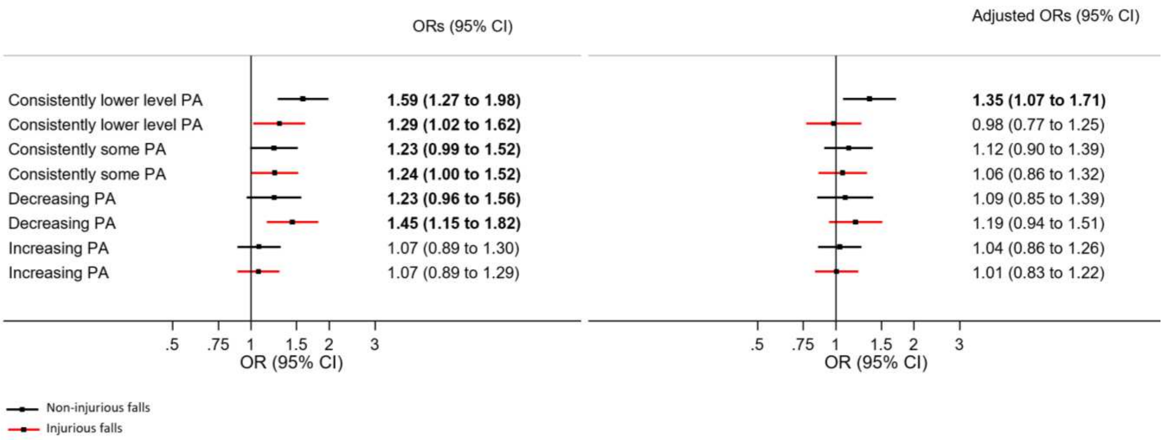

Complete case analysis (n=7,140) to examine the association between the different physical activity participation patterns from middle age (survey 2, 1998, aged 47-52 years) to older age (survey 8, 2016, aged 65-70 years) and subsequent falls and injurious falls (aged 68-73 years) using multinomial logistic regression and presented in odds ratio and 95% confidence intervals (95% CI). We classified complete case as for sample size only included participants as for sample size only included participants who had been assigned with a physical activity participation pattern from latent class analysis, and responses in survey 9 falls, and all the covariates (Accessibility Remoteness Index of Australia scale (ARIA+), body mass index, number of health conditions, education and ability to manage on income) in survey 8. Adjusted odds ratio was adjusted for covariates collected in survey 8.

Kwok WS, *et al. Br J Sports Med* 2024; 58:919–929. doi: 10.1136/bjsports-2024-108262

Supplementary Figure 8 Complete case analysis for the associations between physical activity participation patterns and subsequent falls and injurious falls when adjusted for survey 2 ARIA+, body mass index, number of health conditions, perceived stress (n=7,128)

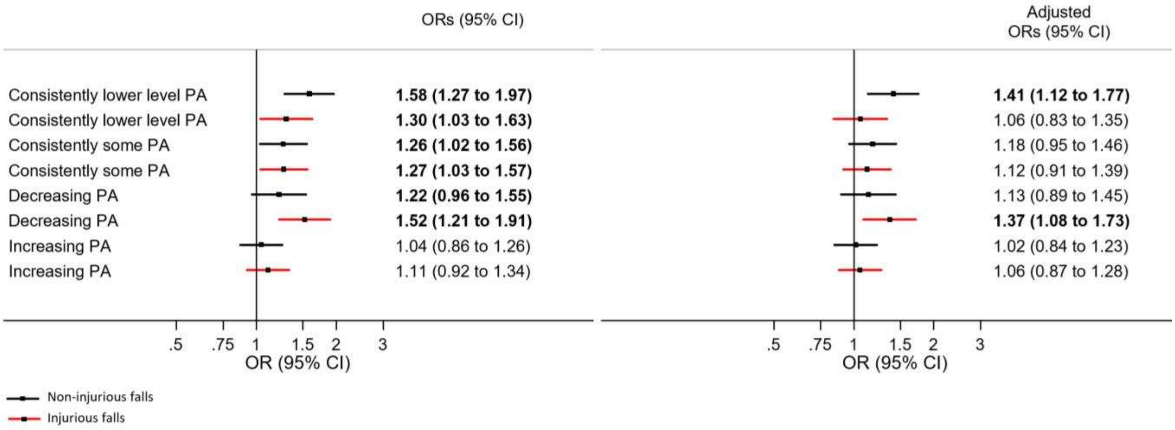

Complete case analysis (n=7,128) to examine the association between the different physical activity participation patterns from middle age (survey 2, 1998, aged 47-52 years) to older age (survey 8, 2016, aged 65-70 years) and subsequent falls and injurious falls (aged 68-73 years) using multinomial logistic regression and presented in odds ratio and 95% confidence intervals (95% CI). We classified complete case as for sample size only included participants who had been assigned with a physical activity participation pattern from latent class analysis, and responses in falls, and all the covariates (Accessibility Remoteness Index of Australia scale (ARIA+), body mass index, number of health conditions, perceived stress) in survey 2. Adjusted odds ratio was adjusted for covariates collected in survey 2.

Supplementary Figure 9 Complete case analysis for the associations between physical activity participation patterns and subsequent falls and injurious falls when adjusted for survey 8 ARIA+, body mass index, number of health conditions, perceived stress (n=7,174)

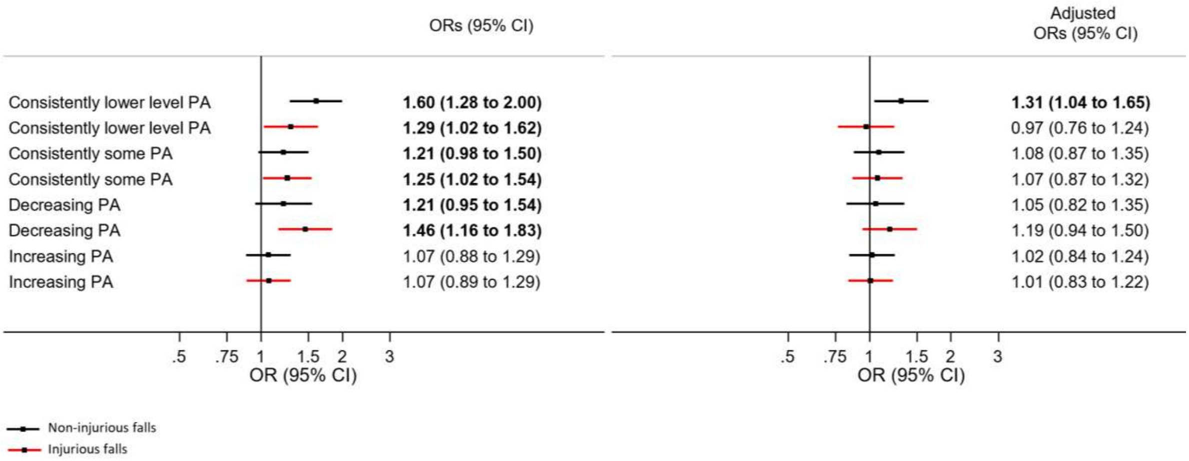

Complete case analysis (n=7,174) to examine the association between the different physical activity participation patterns from middle age (survey 2, 1998, aged 47-52 years) to older age (survey 8, 2016, aged 65-70 years) and subsequent falls and injurious falls (aged 68-73 years) using multinomial logistic regression and presented in odds ratio and 95% confidence intervals (95% CI). We classified complete case as for sample size only included participants who had been assigned with a physical activity participation pattern from latent class analysis, and responses in falls, and all the covariates (Accessibility Remoteness Index of Australia scale (ARIA+), body mass index, number of health conditions, perceived stress) in survey 8. Adjusted odds ratio was adjusted for covariates collected in survey 8.

Kwok WS, *et al. Br J Sports Med* 2024; 58:919–929. doi: 10.1136/bjsports-2024-108262

Appendices References

1. Brown WJ, Burton NW, Marshall AL, Miller YD. Reliability and validity of a modified self-administered version of the Active Australia physical activity survey in a sample of mid-age women. *Australian and New Zealand Journal of Public Health*. 2008;**32**(6):535–41.
2. McCallum J. The SF-36 in an Australian sample: validating a new, generic health status measure. *Australian Journal of Public Health*. 1995;**19**(2):160-6.
3. Australian Longitudinal Study on Women's Health. ALSWH Data Dictionary Supplement Section 5 Geo-coded Data, ARIA Scores: Index of accessibility/remoteness. 2020.
4. Egan N, Forder P, Dobson A, Hockey R. Validation of the ALSWH income management survey question. 2020.
5. Bell S, Lee C, Powers J, Ball J. Multi-item summed score for perceived stress. 2001.
6. van de Schoot R, Sijbrandij M, Winter SD, Depaoli S, Vermunt JK. The GRoLTS-Checklist: Guidelines for Reporting on Latent Trajectory Studies. *Structural equation modeling*. 2017;**24**(3):451-67.
